# Microbial-Derived Daidzin (Eco-3) Suppresses RANKL-Induced Osteoclastogenesis by Targeting the TBK1–NFATc1 Signaling Axis

**DOI:** 10.3390/ijms27177881

**Published:** 2026-09-03

**Authors:** Nivethasri Lakshmana Perumal, Kyung-Bon Koo, Gi-Young Park, Byeong-Churl Jang

**Affiliations:** 1 Department of Molecular Medicine, College of Medicine, Keimyung University, 1095 Dalgubeoldaero, Dalseo-gu, Daegu 42601, Republic of Korea; nivi1998@naver.com; 2 Ecowin Co., Ltd., 18, Techno jungang-daero 2-gil, Yuga-eup, Dalseong-gun, Daegu 42415, Republic of Korea; bon612@hanmail.net; 3 Department of Rehabilitation Medicine, Daegu Catholic University School of Medicine, Nam-Gu, Daegu 42472, Republic of Korea

**Keywords:** Eco-3, microbial-derived daidzin, osteoclastogenesis, TBK1, NFATc1, bone resorption

## Abstract

Eco-3, a microbial-derived daidzin, has been reported to possess diverse biological activities; however, its effects on osteoclast differentiation and the underlying molecular mechanisms remain unclear. In the present study, we investigated the effects of Eco-3 on receptor activator of nuclear factor-κB ligand (RANKL)-induced osteoclastogenesis and explored the signaling pathways involved using RAW264.7 macrophages. Eco-3 suppressed RANKL-induced osteoclast formation at the concentrations tested without significantly affecting cell viability. Eco-3 also altered F-actin organization and reduced the expression of key osteoclastogenic markers, including tartrate-resistant acid phosphatase (TRAP), nuclear factor of activated T cells c1 (NFATc1), c-Fos, and cathepsin K (CTSK). In particular, Eco-3 attenuated NFATc1 expression during the early stage of osteoclast differentiation, suggesting interference with the transcriptional program associated with osteoclastogenesis. Furthermore, RANKL stimulation progressively increased TANK-binding kinase 1 (TBK1) phosphorylation during osteoclast differentiation, whereas Eco-3 attenuated this activation. Genetic silencing of TBK1 suppressed osteoclast formation, while pharmacological inhibition of TBK1 reduced TBK1 phosphorylation, osteoclastogenic gene expression, and osteoclast formation. Collectively, these findings suggest that Eco-3 suppresses RANKL-induced osteoclastogenesis, at least in part, through modulation of the TBK1–NFATc1 signaling axis in RAW264.7 macrophages. Our findings identify TBK1 as a potential regulatory component of osteoclast differentiation and suggest that Eco-3 warrants further investigation as a potential bioactive candidate for osteoclast-related bone disorders.

## 1. Introduction

Bone remodeling is a dynamic physiological process that maintains skeletal integrity through a tightly regulated balance between bone-forming osteoblasts and bone-resorbing osteoclasts [1]. Disruption of this balance, particularly excessive osteoclast activation, contributes to the development and progression of various bone-destructive disorders, including osteoporosis, rheumatoid arthritis, periodontitis, and medication-related osteonecrosis of the jaw [2,3]. Therefore, understanding the molecular mechanisms underlying osteoclast differentiation and function is important for elucidating pathological bone loss and identifying potential therapeutic targets. RANKL-induced osteoclast differentiation is tightly regulated by multiple intracellular signaling pathways that ultimately converge on key transcription factors responsible for osteoclast formation and maturation.

Osteoclasts are multinucleated giant cells derived from cells of the monocyte/macrophage lineage, and their differentiation is primarily driven by receptor activator of nuclear factor-κB ligand (RANKL) signaling [4,5,6]. Upon binding to its receptor RANK, RANKL activates multiple intracellular signaling pathways, including nuclear factor-κB (NF-κB), mitogen-activated protein kinases (MAPKs), phosphatidylinositol 3-kinase (PI3K)/Akt, and calcium-dependent signaling cascades, which converge on nuclear factor of activated T cells cytoplasmic 1 (NFATc1), a master transcription factor governing osteoclastogenesis [7,8,9,10]. Activated NFATc1 subsequently induces the expression of osteoclast-associated genes, including tartrate-resistant acid phosphatase (TRAP), cathepsin K (CTSK), dendritic cell-specific transmembrane protein (DC-STAMP), and other molecules required for osteoclast maturation and bone-resorptive activity [11,12]. Consequently, modulation of NFATc1 signaling represents an important strategy for controlling excessive osteoclast activation and pathological bone resorption.

TANK-binding kinase 1 (TBK1) is a serine/threonine kinase best known for its roles in innate immunity, inflammatory signaling, autophagy, and type I interferon responses [13]. Emerging evidence suggests that TBK1 may also participate in skeletal homeostasis and osteoclast-related signaling. Previous studies have implicated TBK1 in the regulation of RANKL-induced osteoclast differentiation through its interactions with intracellular signaling pathways associated with osteoclastogenesis [14]. However, the precise role of TBK1 in osteoclast differentiation appears to be context-dependent and remains incompletely understood. In particular, the temporal activation of TBK1 during osteoclast differentiation and its functional relationship with NFATc1 require further investigation.

Natural products and their derivatives have attracted considerable attention as potential modulators of osteoclastogenesis because of their diverse biological activities. Accumulating evidence indicates that several bioactive natural compounds can suppress osteoclast differentiation by modulating NFATc1-associated signaling pathways. For example, lumichrome has been reported to inhibit RANKL-induced osteoclastogenesis and bone resorption through suppression of calcium signaling and NFAT activation [15]. These observations support further investigation of bioactive compounds capable of modulating key signaling networks involved in osteoclast differentiation.

Microbial biotransformation provides a useful strategy for generating structurally modified flavonoid and isoflavonoid derivatives with altered or enhanced biological activities [16]. Eco-3 is a microbial-derived daidzin generated through biotransformation by *Xenorhabdus nematophila*. In our previous study, Eco-3 exhibited anti-adipogenic activity by suppressing adipocyte differentiation and lipid accumulation in both cellular and zebrafish models [17]. These findings suggest that Eco-3 can modulate cellular differentiation and metabolic processes. However, its effects on osteoclast differentiation and the underlying molecular mechanisms remain unknown. Based on these observations, we investigated whether Eco-3 modulates RANKL-induced osteoclastogenesis and whether TBK1–NFATc1 signaling is involved in this process.

In the present study, we evaluated the effects of Eco-3 on RANKL-induced osteoclast differentiation using RAW264.7 macrophages and investigated the underlying molecular mechanisms. In particular, we examined TBK1 activation during osteoclastogenesis and its potential relationship with NFATc1 signaling using genetic and pharmacological approaches. Our findings indicate that Eco-3 suppresses RANKL-induced osteoclast differentiation and suggest that modulation of the TBK1–NFATc1 signaling axis may contribute to its anti-osteoclastogenic effects. These findings provide new insights into the regulation of osteoclastogenesis by TBK1 and support further investigation of Eco-3 as a potential bioactive candidate for osteoclast-related bone disorders.

## 2. Results

### 2.1. Establishment of RANKL-Induced Osteoclast Differentiation in RAW264.7 Macrophages

**Figure 1:**
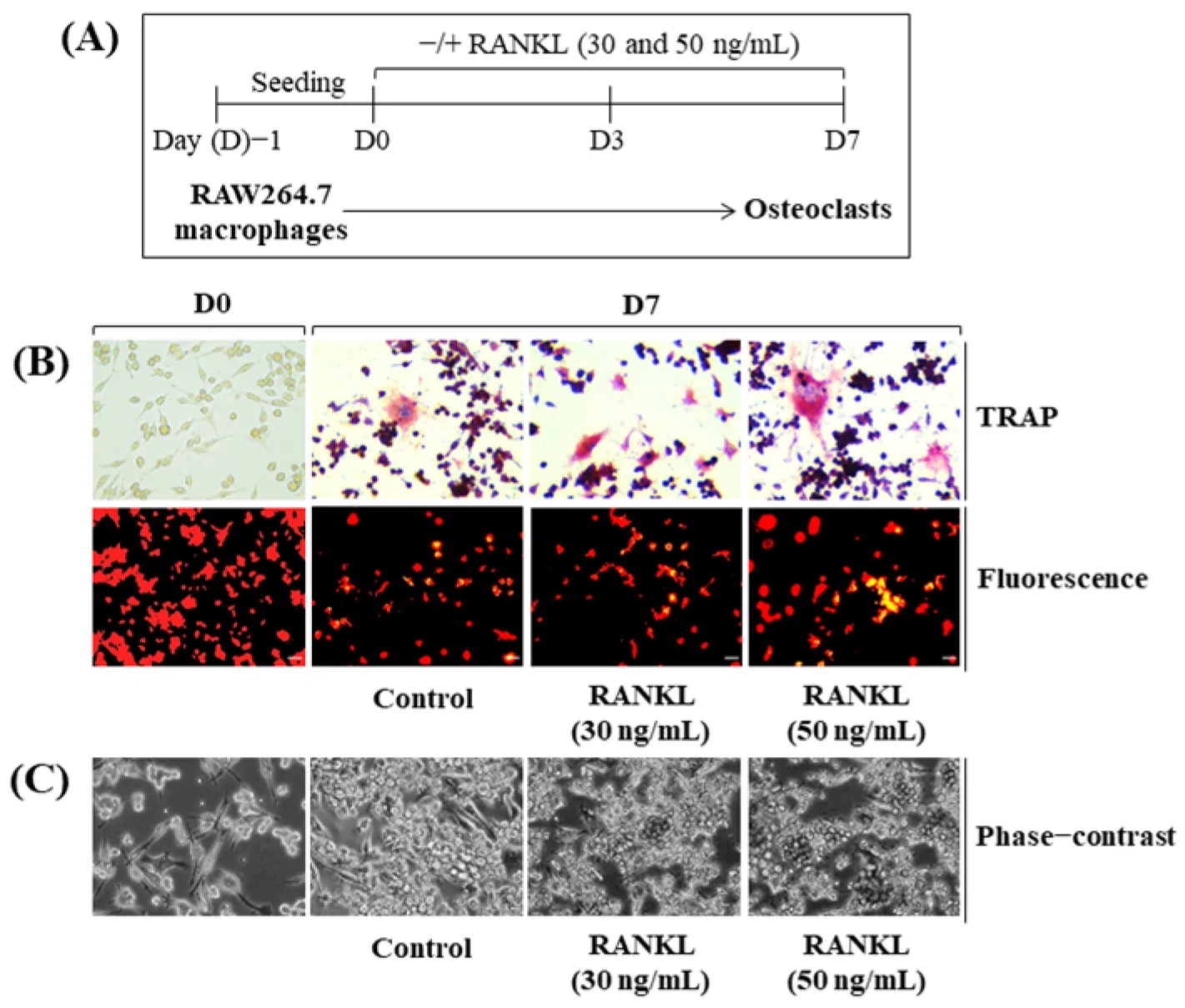
Establishment of RANKL-Induced Osteoclast Differentiation in RAW264.7 Macrophages. (**A**) Experimental design for RANKL-induced osteoclast differentiation. RAW264.7 macrophages were stimulated with recombinant RANKL (30 or 50 ng/mL) for 7 days. (**B**) Representative TRAP staining and TRAP fluorescence images of RAW264.7 macrophages at day 0 (D0) and day 7 (D7) under control and RANKL-treated conditions. (**C**) Representative phase-contrast images showing morphological changes associated with RANKL-induced osteoclast differentiation, including the appearance of multinucleated osteoclast-like cells. Scale bar, 50 μm. Representative images from a single independent experiment (*n* = 1) are shown; no technical replicates were performed.

To establish experimental conditions for RANKL-induced osteoclast differentiation, RAW264.7 macrophages were stimulated with recombinant RANKL (30 or 50 ng/mL) for 7 days according to the experimental scheme shown in Figure 1A. Osteoclast differentiation was evaluated by TRAP staining, fluorescence imaging, and phase-contrast microscopy (Figure 1B,C). At D0, RAW264.7 cells exhibited a typical macrophage-like morphology with minimal TRAP staining. Following RANKL stimulation, morphological and staining changes associated with osteoclast differentiation were observed, including increased cell spreading and the appearance of multinucleated TRAP-positive osteoclast-like cells. These changes were observed at both 30 and 50 ng/mL RANKL, although the representative images did not show a clear morphological difference between the two concentrations. Phase-contrast microscopy similarly showed morphological changes consistent with osteoclast differentiation following RANKL treatment. Additional representative images are provided in Supplementary Figure S1. In addition, the expression of osteoclastogenic markers supported the induction of osteoclast differentiation under the 50 ng/mL RANKL condition (Supplementary Figure S2. Based on these observations and the established use of 50 ng/mL RANKL for inducing osteoclast differentiation in RAW264.7 cells, this concentration was selected as the working condition for subsequent experiments.

### 2.2. Eco-3 Suppresses RANKL-Induced Osteoclast Differentiation Without Significant Cytotoxicity

**Figure 2:**
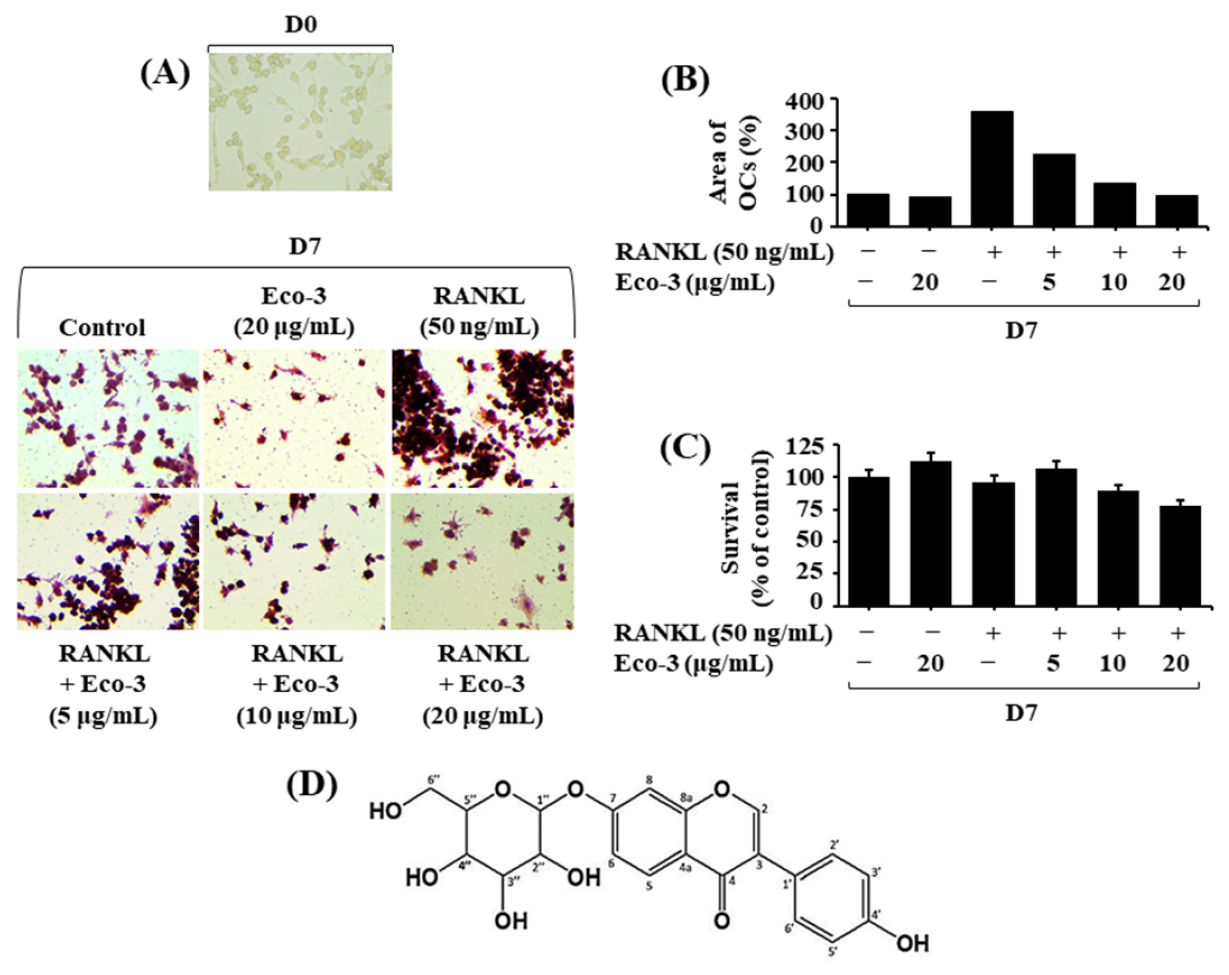
Eco-3 suppresses RANKL-induced osteoclast differentiation without significant cytotoxicity. (**A**) Representative TRAP (tartrate-resistant acid phosphatase)-stained images of RAW264.7 macrophages cultured with RANKL (50 ng/mL) in the presence or absence of Eco-3 (5, 10, and 20 μg/mL) for 7 days. Scale bar, 50 μm. (**B**) Quantitative analysis of TRAP-positive osteoclast area using ImageJ software (v.1.6.0.24; National Institutes of Health, Bethesda, MD, USA). Values are expressed relative to the untreated control, which was set to 100%. (**C**) Cell survival of RAW264.7 macrophages cultured with or without RANKL (50 ng/mL) in the presence or absence of Eco-3 (5, 10, and 20 μg/mL) for 7 days. Data are presented as the mean ± SE from three independent experiments (*n* = 3). Statistical significance was determined by one-way analysis of variance (ANOVA). (**D**) The chemical structure of Eco-3.

To determine the effect of Eco-3 on RANKL-induced osteoclast differentiation, RAW264.7 macrophages were cultured with 50 ng/mL RANKL in the presence or absence of Eco-3 (5, 10, and 20 μg/mL) for 7 days. As shown in Figure 2A, RANKL stimulation promoted the formation of TRAP-positive multinucleated osteoclast-like cells compared with the untreated control. Treatment with Eco-3 reduced RANKL-induced osteoclast formation at the concentrations tested. Quantitative analysis further showed a reduction in TRAP-positive osteoclast area following Eco-3 treatment compared with the RANKL-treated group (Figure 2B). Notably, the inhibitory responses observed at 10 and 20 μg/mL Eco-3 were comparable, indicating that a clear concentration-dependent difference was not evident between these concentrations under the experimental conditions. To determine whether the inhibitory effect of Eco-3 was associated with cytotoxicity, cell survival was initially assessed under the same experimental conditions (Figure 2C). No substantial reduction in cell survival was observed at the tested concentrations of Eco-3. Because cell counting may be influenced by cell fusion during osteoclast differentiation, an additional MTS assay was performed to independently evaluate cell viability. The MTS assay confirmed that Eco-3 did not significantly affect RAW264.7 cell viability at the concentrations tested (Supplementary Figure S3), supporting the conclusion that the observed inhibition of osteoclastogenesis was not attributable to nonspecific cytotoxicity. The chemical structure of Eco-3 is shown in Figure 2D. Based on its inhibitory effect on RANKL-induced osteoclastogenesis, 20 μg/mL Eco-3 was selected as the working concentration for subsequent mechanistic experiments.

### 2.3. Eco-3 Alters F-Actin Organization During RANKL-Induced Osteoclast Differentiation

**Figure 3:**
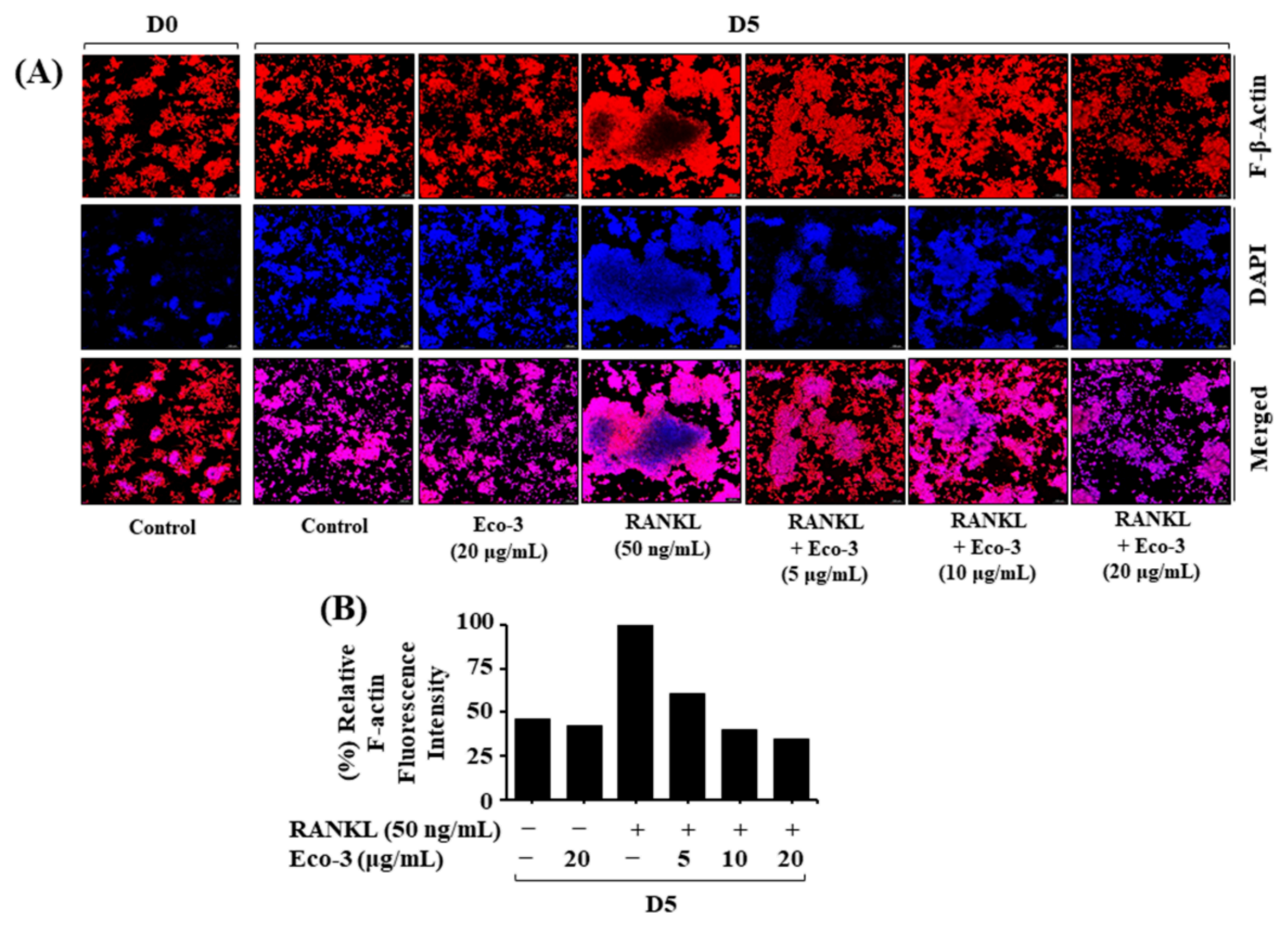
Eco-3 alters F-actin organization during RANKL-induced osteoclast differentiation. (**A**) Representative fluorescence images of F-actin (red), DAPI-stained nuclei (blue), and merged images of RAW264.7 macrophages cultured with RANKL (50 ng/mL) in the presence or absence of Eco-3 (5, 10, and 20 μg/mL) for 5 days. Scale bar, 100 μm. (**B**) Quantitative analysis of relative F-actin fluorescence intensity using ImageJ software. The RANKL-treated group was set to 100% for relative normalization. The quantitative data were obtained from a single independent experiment (*n* = 1) and are presented descriptively without statistical analysis.

Because reorganization of the actin cytoskeleton is associated with osteoclast differentiation and maturation, we next examined the effect of Eco-3 on F-actin organization during RANKL-induced osteoclastogenesis. As shown in Figure 3A, RANKL treatment (50 ng/mL) induced prominent changes in F-actin organization accompanied by the formation of large multinucleated cells compared with the untreated control. Treatment with Eco-3 (5, 10, and 20 μg/mL) altered the F-actin staining patterns observed in RANKL-treated cells, although a clear concentration-dependent pattern was not evident among the Eco-3-treated groups. F-actin staining (red), DAPI-stained nuclei (blue), and merged images were used to visualize cytoskeletal organization and multinucleated cell formation. Quantitative analysis of relative F-actin fluorescence intensity showed lower values in the Eco-3-treated groups than in the RANKL-treated group (Figure 3B). However, because the quantitative analysis was derived from a single independent experiment (*n* = 1), these data are presented descriptively without statistical analysis and should be interpreted cautiously. Additional representative images are provided in Supplementary Table S1. Collectively, these observations suggest that Eco-3 treatment is associated with alterations in F-actin organization during RANKL-induced osteoclast differentiation, although further independent experiments are required to confirm this effect.

### 2.4. Eco-3 Suppresses NFATc1 Expression and Osteoclastogenic Gene Expression

**Figure 4:**
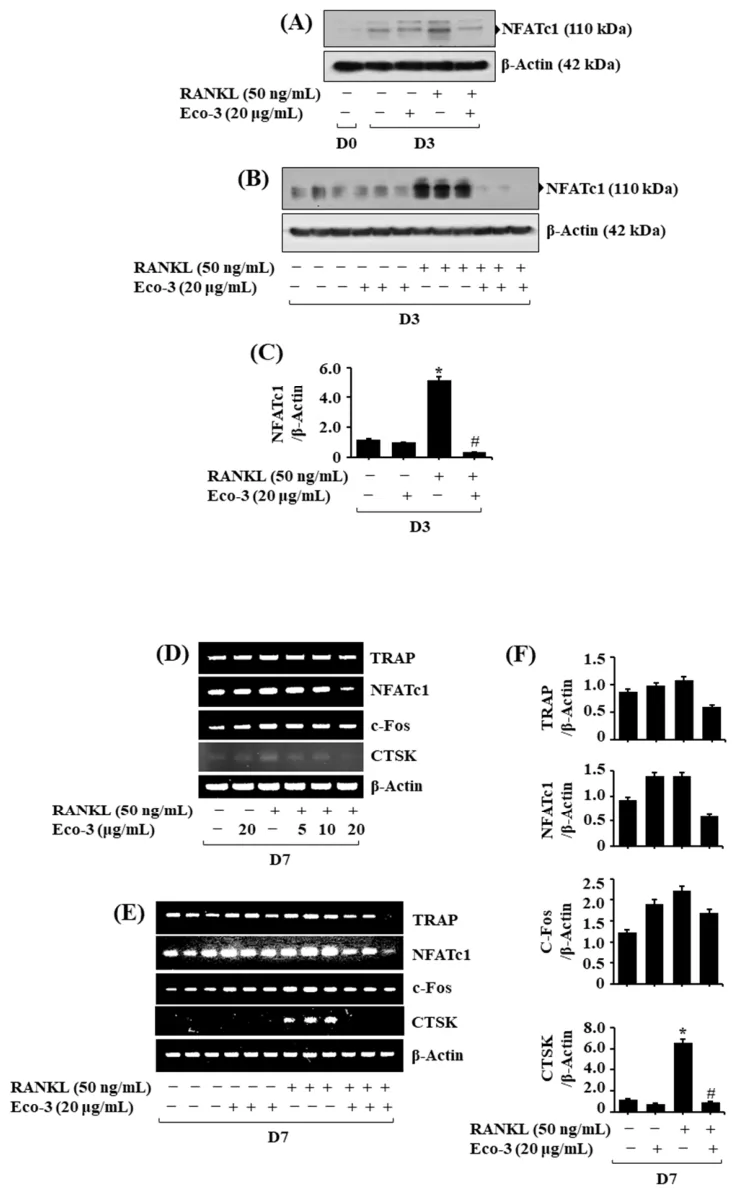
Eco-3 suppresses NFATc1 expression and osteoclastogenic gene expression during RANKL-induced osteoclast differentiation. (**A**) Representative Western blot analysis of NFATc1 protein expression in RAW264.7 macrophages at day 0 (D0) and day 3 (D3) following treatment with RANKL (50 ng/mL) in the presence or absence of Eco-3 (20 μg/mL). (**B**) Western blot analysis of NFATc1 protein expression from three independent experiments under the indicated treatment conditions at D3. (**C**) Densitometric analysis of NFATc1 protein expression normalized to β-actin. (**D**) Representative RT-PCR analysis of TRAP, NFATc1, c-Fos, and CTSK mRNA expression in RAW264.7 macrophages treated with RANKL (50 ng/mL) in the presence or absence of Eco-3 (5, 10, and 20 μg/mL) for 7 days (D7). (**E**) RT-PCR analysis of TRAP, NFATc1, c-Fos, and CTSK mRNA expression from three independent experiments under the indicated treatment conditions with Eco-3 (20 μg/mL) at D7. β-Actin was used as the internal control. (**F**) Densitometric analysis of TRAP, NFATc1, c-Fos, and CTSK mRNA expression normalized to β-actin. Data in (**C**,**F**) are presented as the mean ± SE from three independent experiments (*n* = 3). Statistical significance was determined by one-way ANOVA. * *p* < 0.05 versus the untreated control; # *p* < 0.05 versus the RANKL-treated group.

To investigate the molecular mechanisms underlying the inhibitory effect of Eco-3 on osteoclast differentiation, NFATc1 protein expression was examined during the early stage of RANKL-induced osteoclastogenesis. RAW264.7 macrophages were stimulated with RANKL (50 ng/mL) in the presence or absence of Eco-3, and NFATc1 protein expression was analyzed on day 3 (D3). As shown in Figure 4A, RANKL treatment increased NFATc1 protein expression, whereas treatment with Eco-3 (20 μg/mL) attenuated this induction. This effect was further evaluated in three independent experiments (Figure 4B). Densitometric analysis confirmed that RANKL significantly increased NFATc1 protein expression and that Eco-3 treatment significantly reduced RANKL-induced NFATc1 expression (Figure 4C). To determine whether Eco-3 also affected osteoclastogenic gene expression during the later stage of differentiation, total RNA was isolated from RAW264.7 macrophages cultured with RANKL in the presence or absence of Eco-3 for 7 days, followed by RT-PCR analysis. Eco-3 treatment altered the RANKL-induced expression of osteoclastogenic genes, including TRAP, NFATc1, c-Fos, and CTSK (Figure 4D). These effects were further examined in independent experiments using Eco-3 at 20 μg/mL (Figure 4E). Densitometric analysis showed lower expression levels of these osteoclastogenic genes following Eco-3 treatment, with a particularly pronounced reduction in CTSK expression under the experimental conditions (Figure 4F). Collectively, these findings indicate that Eco-3 attenuates the early induction of NFATc1 protein and subsequently suppresses the expression of osteoclast-associated genes during RANKL-induced osteoclast differentiation.

### 2.5. Eco-3 Attenuates TBK1 Activation During RANKL-Induced Osteoclast Differentiation

**Figure 5:**
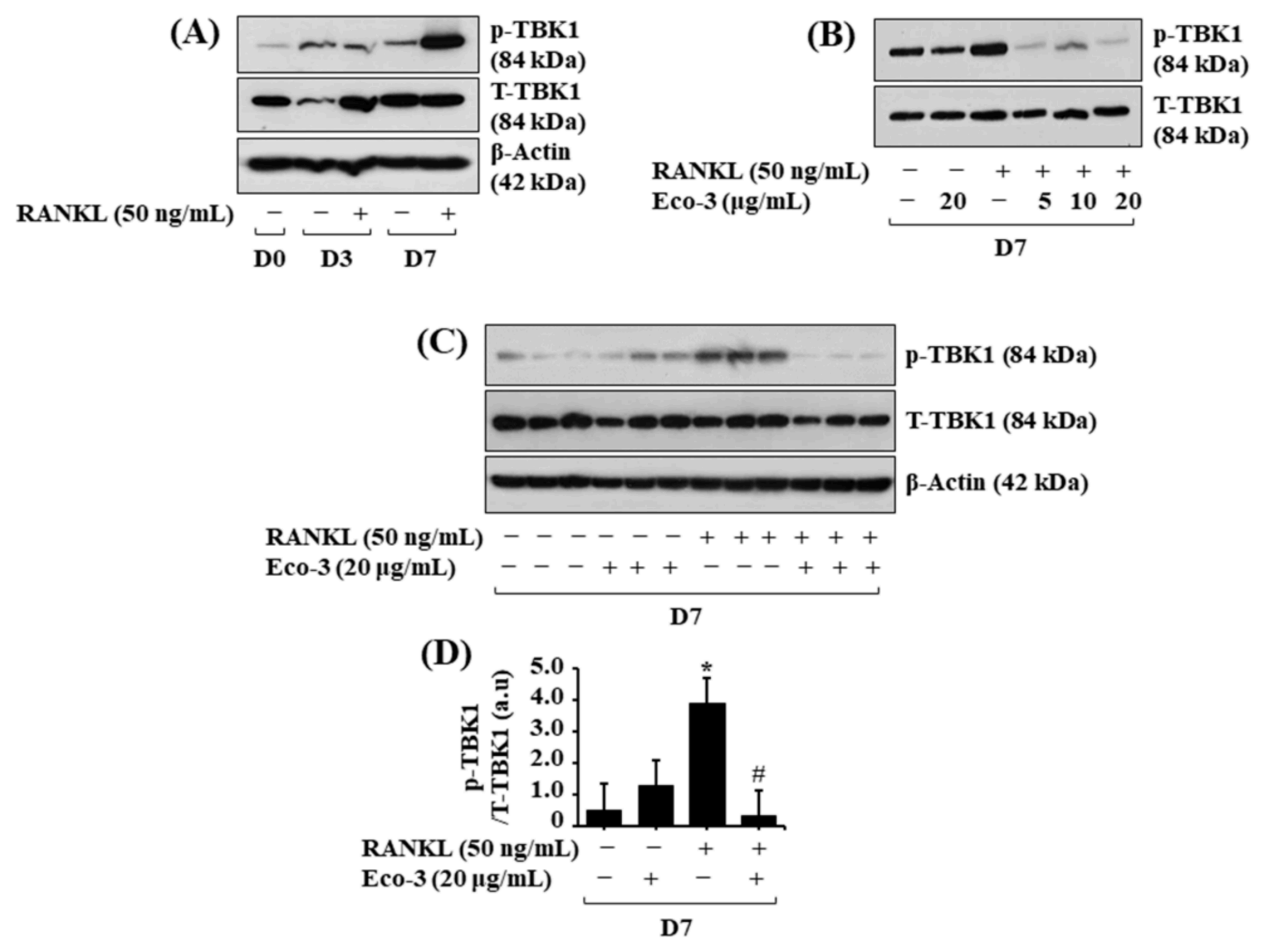
Eco-3 attenuates TBK1 activation during RANKL-induced osteoclast differentiation. (**A**) Representative Western blot analysis of phosphorylated TBK1 (p-TBK1), total TBK1 (T-TBK1), and β-actin in RAW264.7 macrophages cultured in the presence or absence of RANKL (50 ng/mL) for 0 (D0), 3 (D3), and 7 (D7) days. (**B**) Representative Western blot analysis of p-TBK1 and T-TBK1 in RAW264.7 macrophages treated with RANKL (50 ng/mL) in the presence or absence of Eco-3 (5, 10, and 20 μg/mL) for 7 days. (**C**) Western blot analysis of p-TBK1 and T-TBK1 from three independent experiments in RAW264.7 macrophages treated with RANKL (50 ng/mL) in the presence or absence of Eco-3 (20 μg/mL) for 7 days. β-Actin was used as the loading control. (**D**) Densitometric analysis of p-TBK1 normalized to T-TBK1. Data are presented as the mean ± SE from three independent experiments (*n* = 3). Statistical significance was determined by one-way ANOVA. * *p* < 0.05 versus the untreated control; # *p* < 0.05 versus the RANKL-treated group. a.u, arbitrary unit.

To determine whether TBK1 is activated during RANKL-induced osteoclast differentiation, RAW264.7 macrophages were cultured in the presence or absence of RANKL (50 ng/mL), and protein samples were collected on days 0 (D0), 3 (D3), and 7 (D7). Western blot analysis showed that TBK1 phosphorylation increased during osteoclast differentiation, with a prominent increase observed at D7, whereas total TBK1 (T-TBK1) expression remained relatively unchanged (Figure 5A). We next examined whether Eco-3 modulates TBK1 activation during RANKL-induced osteoclastogenesis. At D7, Eco-3 treatment reduced RANKL-induced TBK1 phosphorylation at the concentrations tested, while total TBK1 expression remained relatively unchanged (Figure 5B). This inhibitory effect was further evaluated using Eco-3 at 20 μg/mL in three independent experiments (Figure 5C). Densitometric analysis confirmed that RANKL significantly increased TBK1 phosphorylation and that Eco-3 significantly attenuated this increase (Figure 5D). Collectively, these findings indicate that TBK1 activation is enhanced during RANKL-induced osteoclast differentiation and that Eco-3 attenuates this activation, suggesting an association between TBK1 signaling and the anti-osteoclastogenic effects of Eco-3.

### 2.6. TBK1 Knockdown Suppresses RANKL-Induced Osteoclast Differentiation Without Affecting Cell Survival

**Figure 6:**
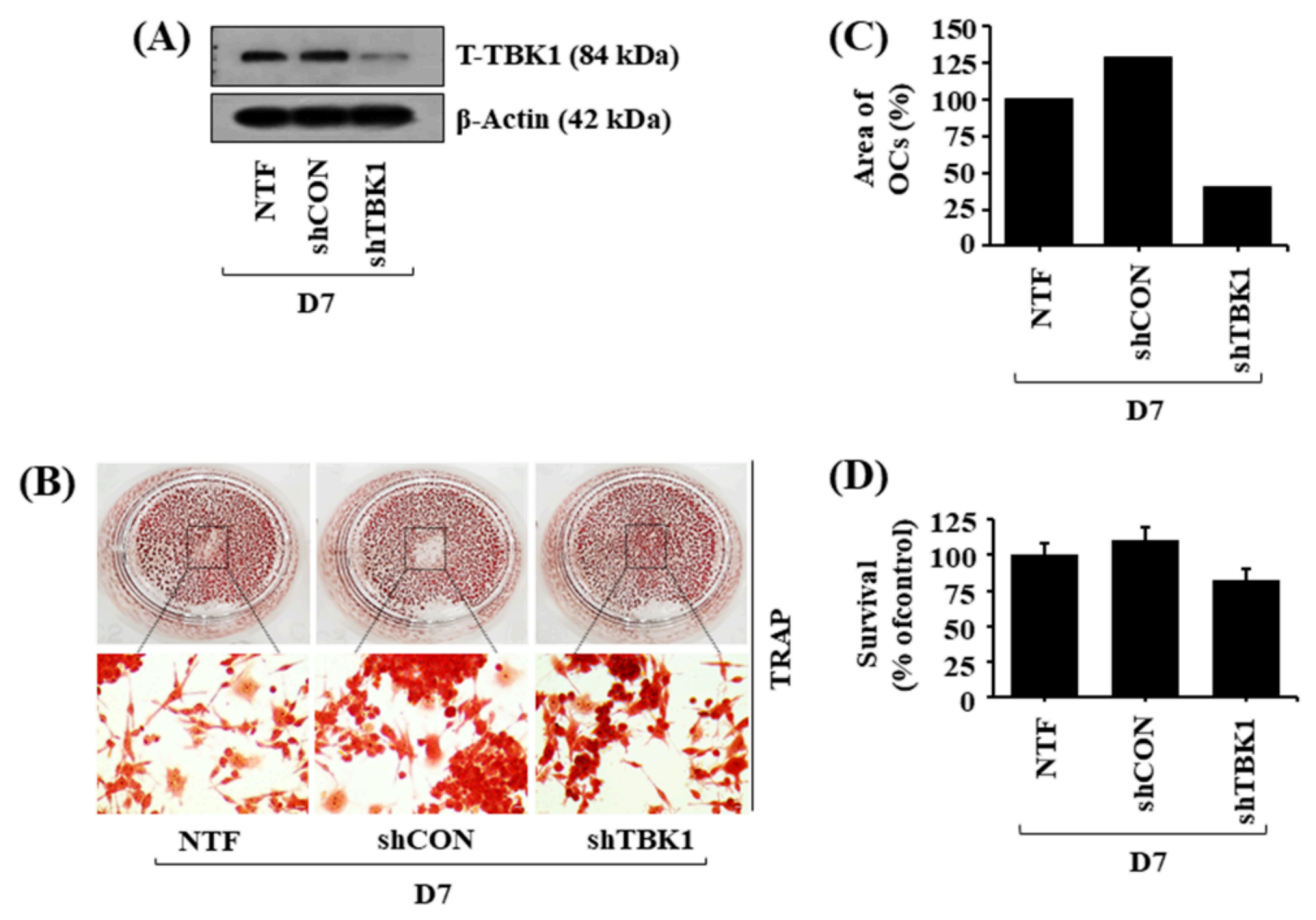
TBK1 knockdown suppresses RANKL-induced osteoclast differentiation without affecting cell survival. (**A**) Representative Western blot analysis confirming TBK1 knockdown in RAW264.7 macrophages transfected with TBK1-specific shRNA (shTBK1). NTF, non-transfected cells; shCON, control shRNA. (**B**) Representative TRAP (tartrate-resistant acid phosphatase)-stained images of osteoclast-like cells in the NTF, shCON, and shTBK1 groups following 7 days (D7) of RANKL-induced osteoclast differentiation. Scale bar, 50 μm. (**C**) Quantitative analysis of the TRAP-positive osteoclast area using ImageJ software. The quantitative data were obtained from a single independent experiment (*n* = 1) and are presented descriptively without statistical analysis. (**D**) Cell survival of NTF, shCON, and shTBK1 RAW264.7 macrophages under the same experimental conditions. The cell survival data are presented as the mean ± SE from three independent experiments (*n* = 3). Statistical significance was determined by one-way ANOVA.

To further investigate the functional role of TBK1 in RANKL-induced osteoclast differentiation, RAW264.7 macrophages were transfected with TBK1-specific shRNA (shTBK1), and osteoclast differentiation was evaluated on day 7 (D7). Western blot analysis confirmed reduced TBK1 protein expression in the shTBK1 group compared with the non-transfected (NTF) and control shRNA (shCON) groups, confirming effective TBK1 knockdown (Figure 6A). The representative triplicate western blotting images are provided in Supplementary Figure S4. To examine the effect of TBK1 depletion on osteoclast differentiation, TRAP staining was performed under the same experimental conditions. As shown in Figure 6B, TRAP-positive osteoclast-like cells were observed in both the NTF and shCON groups following RANKL stimulation, whereas TBK1 knockdown reduced TRAP-positive osteoclast formation. Additional maginifed images are provided in Supplementary Figure S5. Quantitative analysis also showed a lower TRAP-positive osteoclast area in the shTBK1 group compared with the control groups (Figure 6C). To determine whether the inhibitory effect associated with TBK1 knockdown was accompanied by reduced cell survival, cell survival was evaluated under the same experimental conditions. No significant difference in cell survival was observed between the shTBK1 and control groups (Figure 6D), indicating that the reduction in osteoclast formation was not attributable to decreased cell survival. Collectively, these findings indicate that genetic depletion of TBK1 suppresses RANKL-induced osteoclast differentiation without substantially affecting cell survival, supporting a functional role for TBK1 in osteoclastogenesis.

### 2.7. Pharmacological Inhibition of TBK1 Suppresses Osteoclastogenic Gene Expression and Osteoclast Differentiation

**Figure 7:**
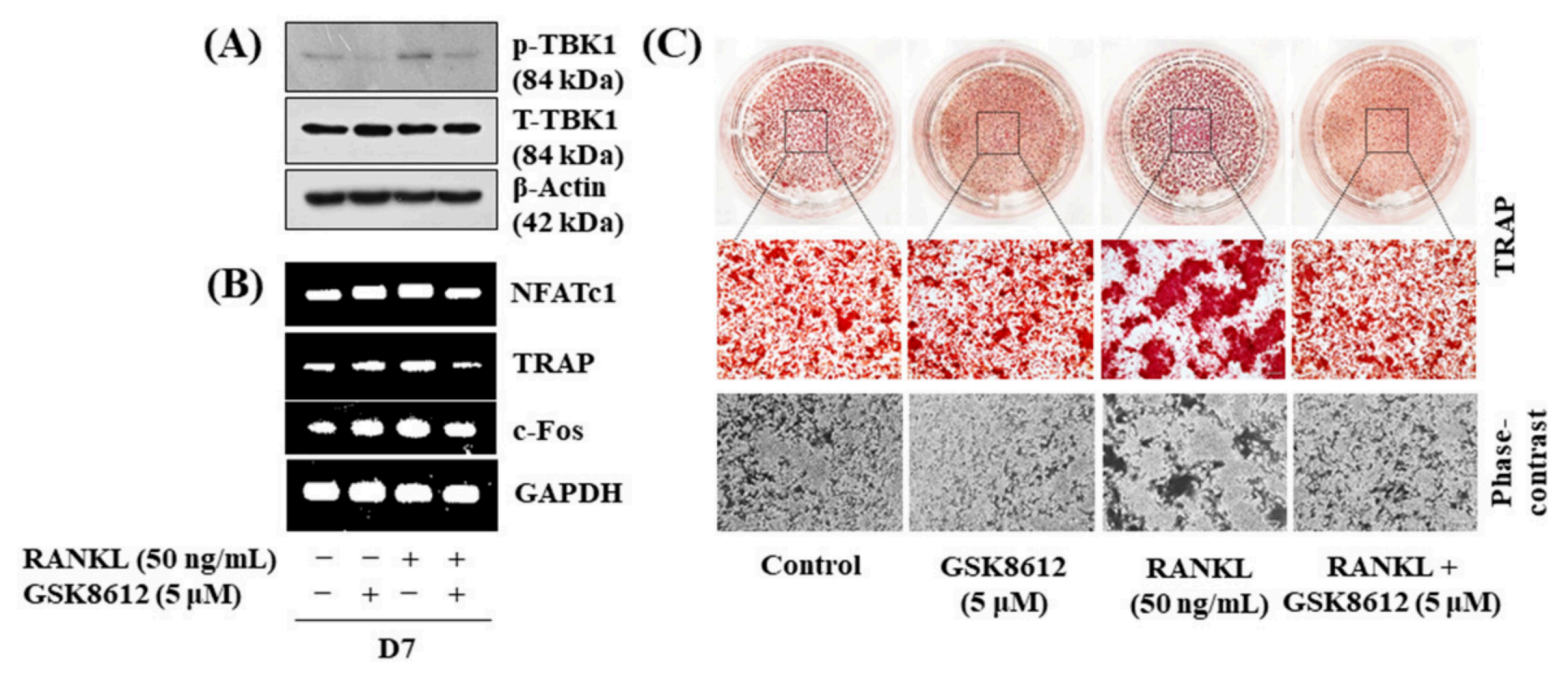
Pharmacological inhibition of TBK1 suppresses osteoclastogenic gene expression and osteoclast differentiation during RANKL-induced osteoclastogenesis. (**A**) Representative Western blot analysis of phosphorylated TBK1 (p-TBK1), total TBK1 (T-TBK1), and β-actin in RAW264.7 macrophages treated with RANKL (50 ng/mL) in the presence or absence of the TBK1 inhibitor GSK8612 (5 μM) for 7 days (D7). (**B**) same experimental conditions. β-Actin was used as the internal control. (**C**) Representative TRAP (tartrate-resistant acid phosphatase)-stained and phase-contrast images of RAW264.7 macrophages cultured with RANKL (50 ng/mL) in the presence or absence of GSK8612 (5 μM) for 7 days. Scale bar, 100 μm. The data shown were obtained from a single independent experiment (*n* = 1) and are presented descriptively without statistical analysis.

To further investigate the functional role of TBK1 in RANKL-induced osteoclast differentiation, RAW264.7 macrophages were treated with the TBK1 inhibitor GSK8612 (5 μM) in the presence or absence of RANKL (50 ng/mL). As shown in Figure 7A, RANKL treatment increased TBK1 phosphorylation (p-TBK1), whereas GSK8612 attenuated RANKL-induced TBK1 phosphorylation, with no apparent change in total TBK1 (T-TBK1) expression. To examine whether pharmacological inhibition of TBK1 affects osteoclastogenic gene expression, RT-PCR analysis was performed under the same experimental conditions. GSK8612 treatment reduced the RANKL-induced mRNA expression of NFATc1, TRAP, and c-Fos (Figure 7B). In contrast, GSK8612 alone showed no apparent effect on the basal expression of these genes in the absence of RANKL. Additional Western blot data on TBK1 phosphorylation are provided in Supplementary Figure S6. The effect of pharmacological TBK1 inhibition on osteoclast differentiation was further examined by TRAP staining. As shown in Figure 7C, RANKL treatment promoted TRAP-positive osteoclast formation, whereas GSK8612 treatment reduced the TRAP-staining phenotype associated with RANKL-induced osteoclast differentiation. Collectively, these observations indicate that pharmacological inhibition of TBK1 attenuates RANKL-induced osteoclastogenic gene expression and osteoclast differentiation, further supporting a functional role for TBK1 signaling in osteoclastogenesis.

## 3. Discussion

The present study provides evidence that Eco-3, a microbial-derived daidzin, suppresses RANKL-induced osteoclast differentiation in RAW264.7 macrophages. Eco-3 reduced TRAP-positive osteoclast formation and altered F-actin organization without significant cytotoxicity. These phenotypic changes were accompanied by reduced expression of NFATc1 and osteoclastogenic markers, including TRAP, c-Fos, and CTSK, suggesting that Eco-3 interferes with the molecular program associated with osteoclast differentiation. Furthermore, Eco-3 attenuated TBK1 phosphorylation during osteoclastogenesis, while genetic knockdown and pharmacological inhibition of TBK1 also suppressed osteoclast differentiation. Together, these findings support a functional association between TBK1 signaling, NFATc1 regulation, and the anti-osteoclastogenic effects of Eco-3.

Microbial biotransformation has emerged as an effective strategy for generating structurally modified natural products with enhanced or novel biological activities [18]. Numerous microbial transformation products derived from flavonoids and isoflavonoids have been reported to exhibit biological activities distinct from or superior to those of their parent compounds [19]. In our previous study, Eco-3, a microbial-derived daidzin, suppressed adipogenesis and lipid accumulation in cellular and zebrafish models through modulation of adipogenic signaling pathways. The present study extends the potential biological activities of Eco-3 to bone metabolism and provides evidence for its previously unrecognized anti-osteoclastogenic activity.

RANKL-induced osteoclastogenesis is orchestrated by multiple intracellular signaling pathways that converge on NFATc1, a master transcription factor governing osteoclast differentiation [20,21]. Upon binding to its receptor RANK on osteoclast precursors, RANKL activates downstream signaling cascades that promote the induction and activation of NFATc1, thereby initiating the transcriptional program required for osteoclast differentiation. Activation of NFATc1 subsequently promotes the expression of osteoclast-associated genes, including TRAP and CTSK, which contribute to osteoclast differentiation, maturation, and bone-resorptive function [22]. In particular, CTSK is a major proteolytic enzyme involved in the degradation of the organic bone matrix, whereas TRAP is widely used as a characteristic marker of differentiated osteoclasts. c-Fos, a component of the activator protein-1 (AP-1) transcription factor complex, also plays a critical role in osteoclastogenesis by facilitating the induction and transcriptional regulation of NFATc1 [23,24]. Thus, coordinated regulation of c-Fos, NFATc1, and their downstream osteoclastogenic genes constitutes a central transcriptional network controlling RANKL-induced osteoclast differentiation and maturation.

Consistent with this established signaling framework, Eco-3 attenuated RANKL-induced NFATc1 protein expression during the early stage of osteoclast differentiation and reduced the expression of osteoclastogenic genes at the later stage. Eco-3 treatment was also associated with alterations in F-actin organization during RANKL-induced osteoclastogenesis. Because cytoskeletal reorganization is an important feature of osteoclast maturation and function, these observations suggest that the effects of Eco-3 may extend beyond regulation of osteoclastogenic gene expression to cellular processes associated with osteoclast maturation. However, because the F-actin analysis was based on a single independent experiment, these observations should be interpreted cautiously and require further validation.

A notable finding of the present study is the potential involvement of TBK1 in RANKL-induced osteoclast differentiation. TBK1 phosphorylation increased during osteoclastogenesis, with prominent activation observed at the later stage of differentiation, whereas Eco-3 attenuated RANKL-induced TBK1 phosphorylation. Importantly, genetic depletion of TBK1 suppressed osteoclast formation without substantially affecting cell survival, providing functional evidence for the involvement of TBK1 in osteoclastogenesis. Pharmacological inhibition of TBK1 using GSK8612 also reduced RANKL-induced TBK1 phosphorylation, osteoclastogenic gene expression, and TRAP-positive osteoclast formation, further supporting this possibility. Together with the observed suppression of NFATc1 by Eco-3, these findings suggest a potential regulatory relationship between TBK1 signaling and NFATc1 during RANKL-induced osteoclast differentiation.

Previous studies have reported that TBK1 can negatively regulate osteoclastogenesis through modulation of the TRAF6–TAK1 signaling pathway or STING-dependent signaling [25]. In contrast, our findings suggest that TBK1 activity may positively contribute to RANKL-induced osteoclast differentiation under the present experimental conditions. These apparently divergent observations highlight the potentially context-dependent role of TBK1 in osteoclast biology. Differences in cellular models, differentiation stages, experimental conditions, or the relative contribution of inflammatory and differentiation-associated signaling pathways may account for these discrepancies. Further studies using primary osteoclast precursors, conditional TBK1-deficient models, and in vivo models of pathological bone loss will therefore be important to define the precise role of TBK1 and its relationship with NFATc1 during osteoclastogenesis.

The present study has several strengths. Osteoclast differentiation was evaluated at multiple levels, including TRAP-positive osteoclast formation, F-actin organization, osteoclastogenic gene expression, and TBK1 signaling. Moreover, the functional involvement of TBK1 was examined using both genetic and pharmacological approaches, providing complementary evidence for its potential role in osteoclastogenesis. Nevertheless, several limitations should be acknowledged. The experiments were performed using the RAW264.7 macrophage model, and the anti-resorptive activity of Eco-3 was not directly assessed using functional bone resorption assays. In addition, several morphological and pharmacological observations were derived from single independent experiments and therefore require further validation. The effects of Eco-3 and TBK1 modulation also remain to be confirmed in primary osteoclast precursors and in vivo models. Furthermore, although our findings suggest a functional relationship between TBK1 and NFATc1, the molecular mechanisms by which TBK1 regulates NFATc1 during osteoclast differentiation remain to be elucidated. Future studies addressing these issues will be important for establishing the physiological and therapeutic relevance of the present findings.

In conclusion, our findings demonstrate that Eco-3 suppresses RANKL-induced osteoclast differentiation and attenuates TBK1 activation and NFATc1-associated osteoclastogenic signaling in RAW264.7 macrophages. Genetic and pharmacological modulation of TBK1 further supports its functional involvement in osteoclastogenesis. These findings identify Eco-3 as a microbial-derived daidzin with anti-osteoclastogenic potential and suggest that the TBK1–NFATc1 signaling relationship may represent a previously underappreciated regulatory mechanism and a potential therapeutic target for disorders associated with excessive osteoclast activity.

## 4. Materials and Methods

### 4.1. Chemicals and Reagents

Recombinant RANKL was purchased from PeproTech (Thermo Fisher Scientific, Rocky Hill, NJ, USA). TRAP staining solution was obtained from Cosmo Bio (Carlsbad, CA, USA). Rhodamine phalloidin was purchased from Cytoskeleton, Inc. (Denver, CO, USA). The Pierce BCA Protein Assay Kit was purchased from Thermo Scientific (Rockford, IL, USA). Enhanced chemiluminescence (ECL) reagent was obtained from Advansta (Menlo Park, CA, USA).

### 4.2. Production and Purification of Eco-3 from Xenorhabdus nematophila

Eco-3 was produced and purified from *Xenorhabdus (X.) nematophila* as previously described [17]. Briefly, *X. nematophila* was cultured in liquid medium at 28 °C with continuous agitation to facilitate the production of microbial metabolites. Following incubation, bacterial cells were removed by centrifugation, and the resulting culture supernatant was collected for metabolite extraction. The supernatant was subjected to organic solvent extraction, and the resulting extract was concentrated under reduced pressure. The crude extract was subsequently fractionated by sequential chromatographic separation using silica gel and reversed-phase C18 columns. The Eco-3-containing fraction was further purified by semi-preparative high-performance liquid chromatography (HPLC), and the purified Eco-3 was used for subsequent experiments.

### 4.3. Osteoclastogenic Induction

RAW264.7 murine macrophages were kindly provided by Professor Jin-Kyung Kim (Department of Microbiology, Keimyung University, School of Medicine, Republic of Korea). The cell line was originally obtained from the American Type Culture Collection (ATCC, Manassas, VA, USA). RAW264.7 cells were cultured in Dulbecco’s Modified Eagle Medium (DMEM; Gibco, Grand Island, NY, USA) supplemented with 10% heat-inactivated fetal bovine serum (FBS; Welgene, Gyeongsan, Republic of Korea) and 1% penicillin–streptomycin. For osteoclast differentiation, cells were seeded at the indicated densities and stimulated with recombinant RANKL (50 ng/mL) in α-minimum essential medium (α-MEM) for up to 7 days. The culture medium was replaced every 2 days. Cells were harvested at the indicated time points (D0, D3, and D7) for subsequent analyses.

### 4.4. TBK1 Short-Hairpin RNA (shRNA)-Mediated Gene Silencing

RAW264.7 cells were plated in 35 mm dishes and maintained overnight to allow for cell attachment. For TBK1 knockdown, cells were transfected with either control shRNA or TBK1-specific shRNA using Lipofectamine™ 2000 (Invitrogen, Carlsbad, CA, USA) according to the manufacturer’s instructions. Following a 6 h transfection period, the transfection medium was replaced with fresh DMEM supplemented with 10% FBS and 1% penicillin–streptomycin. Cells were subsequently maintained for 48 h to allow gene silencing. TBK1 knockdown was confirmed by Western blot analysis, after which osteoclast differentiation was induced with RANKL (50 ng/mL) under the conditions described above.

### 4.5. TRAP Staining and Quantitative Analysis

RAW264.7 macrophages were cultured in 24- or 6-well plates under the indicated experimental conditions. Cells were washed twice with phosphate-buffered saline (PBS), fixed with 4% formaldehyde for 5 min at room temperature, and stained using a commercial tartrate-resistant acid phosphatase (TRAP) staining kit according to the manufacturer’s instructions. The TRAP-positive cells were observed using an inverted microscope (Nikon, Tokyo, Japan). Multinucleated TRAP-positive cells containing three or more nuclei were considered osteoclast-like cells. TRAP-positive osteoclast area was quantified using ImageJ software (v.1.6.0.24; National Institutes of Health, Bethesda, MD, USA) and expressed relative to the indicated reference group.

### 4.6. F-Actin Fluorescence Staining

RAW264.7 cells cultured under the indicated experimental conditions were fixed and permeabilized, followed by incubation with rhodamine phalloidin (100 nM) for 30 min at room temperature in the dark. After washing, nuclei were counterstained with DAPI (1 μg/mL). Fluorescence images were acquired using a confocal laser scanning microscope (STELLARIS 5; Leica Microsystems, Wetzlar, Germany). F-actin-associated fluorescence intensity was quantified using ImageJ software.

### 4.7. Protein Extraction

RAW264.7 cells were washed twice with ice-cold PBS and lysed in modified radioimmunoprecipitation assay (RIPA) buffer containing 50 mM Tris-Cl (pH 7.4), 150 mM NaCl, 0.1% sodium dodecyl sulfate, 0.25% sodium deoxycholate, 1% Triton X-100, 1% Nonidet P-40, 1 mM EDTA, 1 mM EGTA, and a 1× protease inhibitor cocktail. The cell lysates were collected and centrifuged at 13,000 rpm for 15 min at 4 °C. The resulting supernatants were collected, and protein concentrations were determined using the Pierce BCA Protein Assay Kit (Thermo Scientific, Waltham, MA, USA).

### 4.8. Western Blot Analysis

Equal amounts of protein (40 μg) were separated by 10% SDS-PAGE and transferred onto polyvinylidene difluoride (PVDF) membranes (Millipore, Billerica, MA, USA). The membranes were blocked with 5% non-fat dry milk in Tris-buffered saline containing 0.1% Tween-20 (TBST) for 1 h at room temperature and incubated overnight at 4 °C with primary antibodies against NFATc1 (sc-7294; Santa Cruz Biotechnology, Dallas, TX, USA), phospho-TBK1 (Ser172) (Cat. No. 5483; Cell Signaling Technology, Danvers, MA, USA), total TBK1 (Cat. No. 3504; Cell Signaling Technology), and β-actin (A5441; Sigma-Aldrich, St. Louis, MO, USA). After washing with TBST, the membranes were incubated with appropriate horseradish peroxidase (HRP)-conjugated secondary antibodies for 2 h at room temperature. Immunoreactive bands were visualized using enhanced chemiluminescence (ECL) reagents (Advansta, San Jose, CA, USA) and detected using a chemiluminescence imaging system. β-Actin was used as the loading control. Band intensities were quantified using ImageJ software (v.1.6.0.24; National Institutes of Health, Bethesda, MD, USA).

### 4.9. Pharmacological Inhibition of TBK1

To investigate the effect of pharmacological TBK1 inhibition on RANKL-induced osteoclast differentiation, RAW264.7 macrophages were treated with the TBK1 inhibitor GSK8612 (Cat. No. S8872; Selleck Chemicals, Houston, TX, USA) in the presence or absence of RANKL (50 ng/mL). GSK8612 was dissolved in dimethyl sulfoxide (DMSO) to prepare a stock solution and subsequently diluted in culture medium to a final concentration of 5 μM immediately before use. The final concentration of DMSO was maintained at the same level in the corresponding vehicle-control groups. Cells were cultured under the indicated conditions for up to 7 days, and the culture medium containing RANKL and GSK8612 was replaced every 2 days. At the indicated time points, cells were harvested for Western blot and RT-PCR analyses or subjected to TRAP staining as described above.

### 4.10. Semi-Quantitative RT-PCR Analysis

Total RNA was isolated from RAW264.7 macrophages using RNAiso Plus (TaKaRa, Kusatsu, Shiga, Japan) according to the manufacturer’s instructions. RNA concentration and purity were determined using a NanoDrop spectrophotometer (Thermo Fisher Scientific, Waltham, MA, USA). Three micrograms of total RNA were used as the input amount for cDNA synthesis using random hexamer primers and reverse transcriptase. The resulting cDNA was amplified by polymerase chain reaction (PCR) using gene-specific primers for TRAP, NFATc1, c-Fos, CTSK, and β-actin. The primer sequences were as follows: TRAP, forward 5′-CTGGAGTGCACGATGCCAGCGACA-3′ and reverse 5′-TCCGTGCTCGGCGATGGACCAGA-3′; NFATc1, forward 5′-CCGTTGCTTCCAGAAAATAACA-3′ and reverse 5′-TGTGGGATGTGAACTCGGAA-3′; c-Fos, forward 5′-CCAGTCAAGAGCATCAGCAA-3′ and reverse 5′-AAGTAGTGCAGCCCGGAGTA-3′; CTSK, forward 5′-CTTCCAATACGTGCAGCAGA-3′ and reverse 5′-TCTTCAGGGCTTTCTCGTTC-3′; and β-actin, forward 5′-GTCTTTGAGACCTTCAA-3′ and reverse 5′-GTCTTTGCGGATGTCCACG-3′. PCR amplification was performed under the following conditions: TRAP, 35 cycles of denaturation at 95 °C for 20 s, annealing at 69 °C for 20 s, and extension at 72 °C for 30 s; NFATc1, 35 cycles of denaturation at 95 °C for 30 s, annealing at 58 °C for 30 s, and extension at 72 °C for 45 s; c-Fos, 35 cycles of denaturation at 95 °C for 30 s, annealing at 60 °C for 30 s, and extension at 72 °C for 30 s; CTSK, 30 cycles of denaturation at 95 °C for 30 s, annealing at 59 °C for 30 s, and extension at 72 °C for 30 s; and β-actin, 25 cycles of denaturation at 95 °C for 30 s, annealing at 56 °C for 30 s, and extension at 72 °C for 30 s. PCR products were separated by agarose gel electrophoresis, visualized under ultraviolet illumination, and photographed using a gel documentation system. Band intensities were quantified using ImageJ software (v.1.6.0.24; National Institutes of Health, Bethesda, MD, USA) and normalized to β-actin expression.

### 4.11. Cell Survival Assay

RAW264.7 macrophages were plated in 24-well plates and maintained under the specified experimental conditions. To evaluate the potential effects of Eco-3 on cell viability during osteoclast differentiation, cells were treated with Eco-3 in the presence or absence of RANKL for 7 days. On day 7 (D7), cell viability was determined using the trypan blue exclusion assay. Cells with intact plasma membranes remained unstained and were considered viable, whereas cells with damaged membranes absorbed the dye and were identified as non-viable. Viable and non-viable cells were counted using a light microscope. All experiments were performed in triplicate, and data are expressed as the mean ± standard error (SE) of three independent experiments.

### 4.12. Statistical Analysis

Statistical analyses were performed only for experiments with three independent biological replicates (*n* = 3). Data from these experiments are presented as the mean ± standard error (SE). Statistical significance was evaluated using one-way analysis of variance (ANOVA) followed by Dunnett’s multiple comparison test using SPSS Statistics version 11.5 (SPSS Inc., Chicago, IL, USA). Differences were considered statistically significant at *p* < 0.05. Data obtained from a single independent experiment (*n* = 1) are presented descriptively without error bars or statistical analysis. The number of independent experiments performed for each analysis is specified in the corresponding figure legend.

## Data Availability

The data that support the findings of this study are available from the corresponding author upon reasonable request.

## Supplementary Materials

The following supporting information can be downloaded at: https://www.mdpi.com/article/10.3390/ijms27177881/s1.
